# 3D in vivo dosimetry of HDR gynecological brachytherapy using micro silica bead TLDs

**DOI:** 10.1002/acm2.13729

**Published:** 2022-08-10

**Authors:** Ramin Jaberi, Somayyeh Babaloui, Zahra Siavashpour, Maryam Moshtaghi, Alireza Shirazi, Musa Joya, Mohammad Hadi Gholami, Shakardokht Jafari

**Affiliations:** ^1^ Radiation Oncology Department Yas Hospital Complex, Tehran University of Medical Sciences Tehran Iran; ^2^ Department of Physics University of Surrey Guildford UK; ^3^ Department of Medical Physics and Biomedical Engineering Faculty of Medicine Tehran University of Medical Sciences Tehran Iran; ^4^ Radiotherapy Oncology Department, Shohada‐e‐Tajrish Educational Hospital, Medical School Shahid Beheshti University of Medical Sciences Tehran Iran; ^5^ Radiology Department Kabul University of Medical Sciences Afghanistan; ^6^ Department of Medical Radiation Engineering Science and Research Branch, Islamic Azad University Tehran Iran; ^7^ Mahdieh Radiotherapy Oncology Center Hamedan Iran; ^8^ Medical Physics Dept. Portsmouth Hospitals University NHS Trust Portsmouth UK; ^9^ Medical Research Centre Kateb University Kabul Afghanistan

**Keywords:** 3D in vivo dosimetry, GYN, HDR brachytherapy, micro silica bead TLD

## Abstract

**Purpose:**

This study aimed to evaluate the feasibility of defining an in vivo dosimetry (IVD) protocol as a patient‐specific quality assurance (PSQA) using the bead thermoluminescent dosimeters (TLDs) for point and 3D IVD during brachytherapy (BT) of gynecological (GYN) cancer using ^60^Co high‐dose‐rate (HDR) source.

**Methods:**

The 3D in vivo absorbed dose verification within the rectum and bladder as organs‐at‐risk was performed by bead TLDs for 30 GYN cancer patients. For rectal wall dosimetry, 80 TLDs were placed in axial arrangements around a rectal tube covered with a layer of gel. Ten beads were placed inside the Foley catheter to get the bladder‐absorbed dose. Beads TLDs were localized and defined as control points in the treatment planning system (TPS) using CT images of the patients. Patients were planned and treated using the routine BT protocol. The experimentally obtained absorbed dose map of the rectal wall and the point dose of the bladder were compared to the TPSs predicted absorbed dose at these control points.

**Results:**

Relative difference between TPS and TLDs results were −8.3% ± 19.5% and −7.2% ± 14.6% (1SD) for rectum‐ and bladder‐absorbed dose, respectively. Gamma analysis was used to compare the calculated with the measured absorbed dose maps. Mean gamma passing rates of 84.1%, 90.8%, and 92.5% using the criteria of 3%/2 mm, 3%/3 mm, and 4%/2 mm were obtained, respectively. Eventually, a “considering level” of at least 85% as pass rate with 4%/2‐mm criteria was recommended.

**Conclusions:**

A 3D IVD protocol employing bead TLDs was presented to measure absorbed doses delivered to the rectum and bladder during GYN HDR‐BT as a reliable PSQA method. 3D rectal absorbed dose measurements were performed. Differences between experimentally measured and planned absorbed dose maps were presented in the form of a gamma index, which may be used as a warning for corrective action.

## 1 INTRODUCTION

High‐dose‐rate (HDR) image‐guided adaptive brachytherapy (BT) is used to treat cervical and endometrial malignancies as a definitive and adjuvant treatment. This method allows for treatment individualization and plans optimization. Having an accurate three‐dimensional image sequence of the tumor and normal tissue anatomy, their relative locations, and the cancer invasion site could aid in choosing the optimum treatment for each BT session.^1^ The critical characteristics of BT are a steep dose gradient and enhanced dose conformity to the target, which makes it easier to give high absorbed doses to the target while sparing healthy surrounding tissues. However, if high precision is not achieved, this treatment can be a dubious benefit because it exposes the target to a large absorbed dose while simultaneously exceeding the dose limitations of the organs‐at‐risk (OARs).^2^

Therefore, HDR‐BT accuracy has been a great challenge due to the high gradient dose distribution, high user‐dependency of optimization techniques, and vicinity of OARs to the high dose regions and the related intra‐fractional uncertainties.^3^, ^4^, ^5^, ^6^ Hence, it is essential to utilize strategies for an in vivo verification of delivered absorbed dose distributions. Implementation of in vivo dosimetry (IVD) provides information that can minimize the risk of normal tissue complications.

IVD is a radiation measurement taken while the patient is being treated and contains information about the absorbed dose. IVD is the practical method for treatment quality control to specify the treatment procedure accuracy and assess the absorbed dose delivery.^4^, ^7^, ^8^, ^9^ IVD has been used in BT by a variety of dosimeters and measurement technologies, such as thermoluminescent dosimeters (TLDs),^7^, ^10^ diodes,^11^, ^12^ MOSFETs,^3^, ^13^ and plastic scintillation detectors.^14^ Thermoluminescent dosimetry has been determined as a suitable system for dosimetry in BT.

Micro silica bead TLDs, which were discovered in 2014 as novel radiation detectors, exhibit several favorable characteristics; they have a small size and chemically inert nature, are inexpensive, are reusable, have a fading rate of 10% at 30 days after irradiation, high thermoluminescence (TL) light transparency, and an extensive dynamic dose–response range that remains linear (*R* ^2^ ≥ 0.999) from 1 cGy to 25 Gy.^15^, ^16^, ^17^, ^18^, ^19^ Some common TLDs types, such as lithium fluoride (LiF) and dysprosium‐doped calcium sulfate (CaSO_4_:Dy) ones, can only measure absorbed doses of up to 10 Gy^20^ or possess a 28‐day fading rate of 25%–60%.^21^ In addition, the spherical shape of the bead TLDs with a hole in the middle enables them to arrange in 2D and 3D configurations. It makes them applicable for having high‐resolution point absorbed dose measurements or even obtaining 3D absorbed dose distributions.

There are some commercially available dosimetry systems for BT IVD with passive and off‐line reports or active and real‐time absorbed dose monitoring ability.^21^ However, most of these dosimeters report point or linear absorbed doses in the addressed organs. Therefore, having a clinically available and feasible protocol for IVD for double‐checking the delivered dose distribution, especially around the critical OARs, will be an attractive and valuable treatment quality assurance for every BT department.

This study aimed to evaluate the feasibility of defining an IVD protocol as a patient‐specific quality assurance (PSQA) using the bead TLDs for point and 3D IVD during BT of gynecological (GYN) cancer using ^60^Co HDR source. The practical applicability of the defined protocol has also been tested as a pilot study for real GYN cases.

## 2 METHODS

The first phase of the feasibility study of using bead TLDs in BT 3D dosimetry was performed on a female pelvis phantom and has been reported by Babaloui et al.^22^ During the current study, 30 cervical cancer patients who had an indication for HDR‐BT were selected and involved nonrandomly. The previously published IVD protocol for rectum and bladder dose measurements was employed throughout the included patients’ BT sessions at the radiotherapy department of Yas Hospital in Tehran, Iran, between May and December 2019. The primary exclusion criteria for the patient selection were the patient's and her physician's satisfaction. Patient characteristics are presented in Table 1.

**TABLE 1:** Descriptive patient characteristics

No. of patients	30
Age (years)	
Mean	55.5
Range	35–88
Histology: Cervical squamous cell carcinoma 24	
Cervical adenocarcinoma	6
International Federation of Gynecology and Obstetrics (FIGO) stage:	
IA	1
IIA	2
IIB	13
IIIA	3
IIIB	2
IIIC	4
IVA	5

### 2.1 Informed consent

The Tehran University of Medical Sciences (TUMS) review board approved the study protocols (code of ethics: IR.TUMS.MEDICINE.REC.1396.4851). All patients were informed of the study's purposes and written informed consent was obtained from all subjects before participation.

### 2.2 Micro silica bead TL dosimetry process

A batch of 300 bead TLDs (TRUEinvivo Ltd, UK) with a 1.6‐mm diameter and 1.1‐mm thick outer shell was used. The TLDs material compositions include (by weight) O‐57.9%, Na‐12.6%, F‐0.46%, Al‐1.63%, Si‐25.28%, K‐0.65%, Ca‐1.48%, and effective atomic number of 11.03. Jafari et al.^15^, ^16^ published the preparation and annealing procedure for bead TLDs. The element correction coefficient of silica beads was determined using a 6‐MV photon beam (TPR20/10 = 0.67) of a linac (Elekta Compact, Sweden).

The beads were scanned using a Fimel LTM TLD reader (PTW FREIBURG, Germany). The readout cycle begins when the planchet temperature reaches 160°C for 10 s (preheat), followed by maximum heating to 350°C for 12 s at a ramp rate of 25°C/s. To guarantee that the beads were stable before the subsequent irradiation, they were annealed for 1 h at 400°C, then 16 h at 80°C, and lastly, 24 h at room temperature in the dark.

### 2.3 Bladder Foley catheter and rectal tube preparation

**FIGURE 1:**
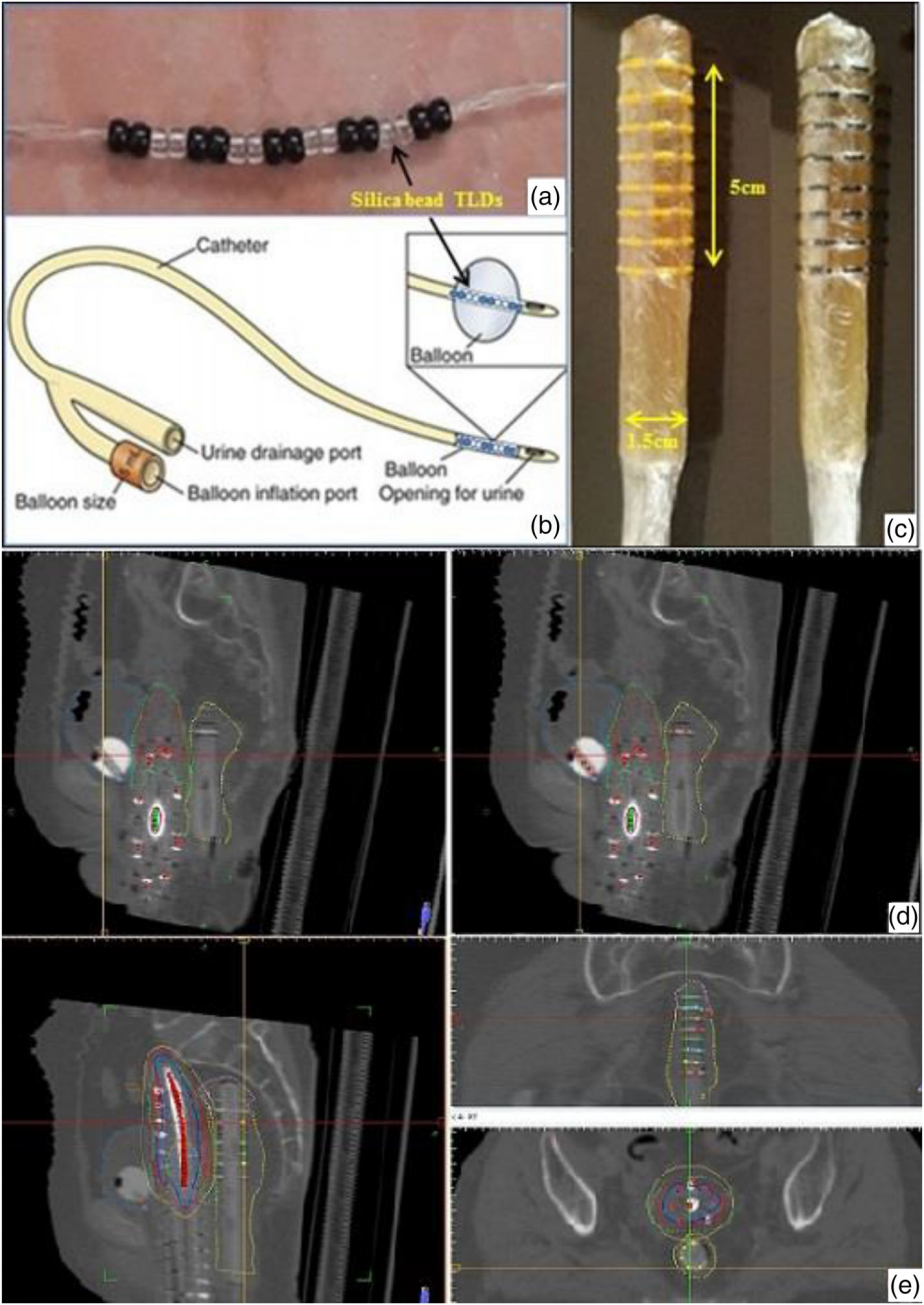
(a) The bead thermoluminescent dosimeters (TLDs) arrangement in a balloon of a Foley catheter, (b) the schematic diagram of the Foley catheter, (c) the prepared rectal tube with the gel coverage and the bead TLDs arrangement covered with some layer of cling film, (d) CT‐scan images of bead TLDs in the bladder Foley catheter, (e) CT‐scan images of bead TLDs on the rectal tube

IVD was performed on 30 patients who received HDR GYN BT followed by external radiation (EBRT). Each patient was only investigated once. A bladder Foley catheter with a French size of 18 was used for all the understudied cases. Ten activated calibrated TLDs and 10 color‐coded marker beads were placed linearly and in alternated pairs inside a Foley catheter balloon to measure the bladder absorbed dose (Figure 1a,b). After the addition of TLDs, the whole Foley catheter was sterilized and inserted into the bladder before the applicator insertion in the operation room. As a basic experimental evaluation, TLD response variation was investigated before and after Foley catheter sterilization, but no significant variation in TLD results was found. As part of our usual bladder preparation, we filled the bladder balloon with 1 cm^3^ of meglumine compound and 6 cm^3^ of distilled water to help visualize the Foley catheter. However, a combination of 0.5 cm^3^ of meglumine compound and 6.5 cm^3^ of distilled water was chosen to achieve a more dilute solution and avoid the beads being masked during the current study. After the Foley catheter insertion and filling, it was pulled out carefully to ensure that the balloon was attached to the bladder neck.

The inner rectal diameter of 20 individuals was assessed randomly on their pelvic CT images before creating the dedicated rectal balloon for rectal absorbed dose assessment with the bead TLDs, and a 1.5‐cm diameter was obtained as the average of these values. As a result, a rectal tube that fills the inside of the rectum without bringing the rectum closer to the applicators was attempted.

The rectal absorbed dose was measured using 80 calibrated bead TLDs arranged axially around a rectal tube (in eight rounds) with a French size of 30 covered with a layer of gel. The bead TLDs were strung on plastic thread and placed in eight rows on the customized rectal tube. Each raw contained 10 TLDs, 2 of which were adhered together, and 4 colored/marker beads were positioned between them. The gel‐coated rectal tube was about 1.5 cm in diameter, and the rectal tube with the beads was around 5‐cm long. Figure 1c shows how the bead rows were spaced 7 mm apart. A layer of cling film was placed over the collection. A condom was placed over the group after the applicators were implanted, and the rectal tube holding the beads was inserted into the rectum in the operating room. The rectal tube was fixed and marked at the anus to reduce its probable displacements, and the Foley catheter was attached to the body.

### 2.4 Implantation, simulation, planning, and HDR‐BT

The insertions were performed with tandem–cylinder–needle or tandem–ovoid (one patient only) GYN applicators (Eckert & Ziegler GmbH, Germany). The implantation procedure took place under local anesthesia (i.e., lumbar puncture). Under abdominal ultrasound guidance, a tandem applicator was placed. A pelvic CT scan (with a slice thickness of 2.5 mm and a pitch of 1.0) was then obtained (GE HiSpeed CT/e Dual Slice CT, USA). Overall, 120 kVp, 80–100 mAs was the CT‐scan acquisition protocol. To better see the beads, the image series were then reconstructed to 0.5‐mm slice thickness. Dummy markers were placed into the needles before the CT simulation.

The CT scans were sent to the treatment planning system (TPS) (HDRplus, v3.0.8, Eckert & Ziegler GmbH, Germany), and the applicator/catheters paths were reconstructed. High‐risk clinical target volume (CTV_HR_), intermediate‐risk clinical target volume (CTV_IR_), and OARs were delineated in the CT images. Automatic treatment planning followed by manual optimization was done to deliver the prescribed dose to the targets while not exceeding the OARs’ dose constraints. After 45–50.4 Gy in 25–28 fractions of EBRT, the HDR‐BT dose prescribed per fraction was roughly 5.5–10.4 Gy, performed in 3 fractions (except for 1 patient who was treated in 2 fractions). The applicators were connected to the afterloader with the transference tubes of the BT machine (MultiSource afterloader, Eckert & Ziegler GmbH, Germany), and treatment was started after a radiation oncologist approved the treatment plan. The average time interval from taking CT images to organs and targets contouring, treatment planning, and performing the treatment ranged from 60 to 90 min. Once a week, HDR‐BT fractions were administered, with new applicator insertion for each treatment fraction.

### 2.5 Data extraction and analysis

The TLDs were visible and localized in TPS as control points on the CT images getting help from the color beads (Figure 1d,e). Absorbed dose to these control points was obtained and extracted after identifying the 3D position of each bead TLD on the CT images and treatment planning final approval. After that, the TPS's estimated absorbed dose to the TLDs surrogate control points in the Foley catheter was compared to the absorbed dose obtained experimentally from TLDs’ measurements. Another test was conducted to see if it was necessary to decide each individual bead as a distinct control point or if we could average the results of two neighboring beads by allocating one control point on each of the two attached TLDs.

A rectal wall dose map was obtained from the interpolating bead TLDs and TPS results. Furthermore, the experimentally acquired absorbed dose map of the rectal wall was compared with the calculated ones by the TPS for each patient using myQA Patients software (IBA Dosimetry, Germany). Gamma analysis was also performed. Gamma analysis is a reliable method that has been used in external beam radiotherapy^23^, ^24^ for the patient or machine‐specific quality assurance. However, the application of gamma index criteria in BT is limited, and the existing investigations were almost only phantom studies.^25^ Having a large number of bead TLDs was an encouraging factor for being able to have high‐resolution IVD in the current study. Therefore, it was tried to use gamma analysis for dose‐ and distance‐to‐agreement (DTA) assessment for the obtained results from the TPS dose distribution and the experimental results from the TLDs array. A DTA of 3.0 mm was considered an acceptable tolerance value for BT audit assessment with a film dosimeter by Palmer et al.^26^ In the current study, glass bead TLDs were employed as a new BT dosimeter during real patient BT. Therefore, gamma criteria of 3% (global normalization)/2 mm, 3%/3 mm, and 4%/2 mm were used to analyze 3D IVD and find the best criteria.

The RT‐Dose and RT‐Image DICOM matrix, including absorbed dose and image information of each case, were exported from the TPS and imported into the MATLAB software (R2019b, MathWorks, US) for further processing. TLDs control points indicator had about 2‐mm diameters. Therefore, RT‐Dose matrix pixel size was averaged to rich the physical size of TLDs and their control points and decrease the uncertainties in later absorbed dose comparison. Three RT‐Dose matrices were evaluated, with 2‐, 3‐, and 4‐mm pixel sizes. Two dose matrices were eventually imported into the myQA Patients software, one extracted from TPS, and the other was obtained from the dosimeter's readouts from experimental measurements.

### 2.6 Measurement uncertainty

In order to obtain reliable results, the assessment of possible absorbed dose measurement uncertainties due to bead TLDs calibration and dose–response curve acquirement are presented in Table 2. The uncertainty in the TLDs readout procedure is affected by the annealing of silica beads, the inconsistency of beads positioning at the planchet, and the fading effect. The uncertainty for the bead measurements was estimated as 10.4% at *k* = 2. Furthermore, based on previously published data, absorbed dose rate uncertainties in a single IVD measurement for individual source dwell positions can range from 3% to 26% (8), and dosimeter positioning errors can produce dosage variances of up to 200% in BT for each measurement point (4).

**TABLE 2:** The uncertainty budget of micro silica bead thermoluminescent dosimeters (TLDs)

Source of uncertainty	Uncertainty (%)	Type
Individual sensitivity correction factors for each silica bead^a^	2.7	A
Dose–response linearity of silica bead TLD^b^	0.1	B
TLD reading process^c^	2.0	A
Silica bead calibration^d^	0.5	B
Energy dependency of the silica beads^e^	1	B
^60^Co source calibration accuracy^f^	3	B
6‐MV photon beam absolute dosimetry calibration^g^	2	B
Determining the position of the bead TLD on TPS^h^	1	A
Calculated combined standard uncertainty (quadratic summation) (*k* = 1)	5.2	
Expanded uncertainty for silica beads (*k* = 2)	10.4	

Abbreviation: TPS, treatment planning system.

^a^ From typical standard deviation of TL signal values of individual beads derived from the response of each dosimeter to the same dose irradiated in a flat homogenous beam.

^b^ Consistency of beam output variations.

^c^ Consistency of TLD reader during the readout process.

^d^ Consistency of calibration setup.

^e^ Energy dependency of the silica beads arising from calibration in 6 MV and measurement in ^60^Co.

^f^ ^60^Co calibration in terms of source strength.

^g^ 6‐MV photon beam absolute dosimetry calibration relating to the uncertainty of the thimble chamber calibration factor, its location, and reading.

^h^ Based on the point diameter of the bead TLD indicator and the pixel size of the image.

## 3 RESULTS

Due to practical concerns, such as rectal catheter and bladder Foley displacements, rectal and bladder absorbed dose measurements were only investigated and analyzed in 29 and 25 patients, respectively, from the 30 selected cases.

**FIGURE 2:**
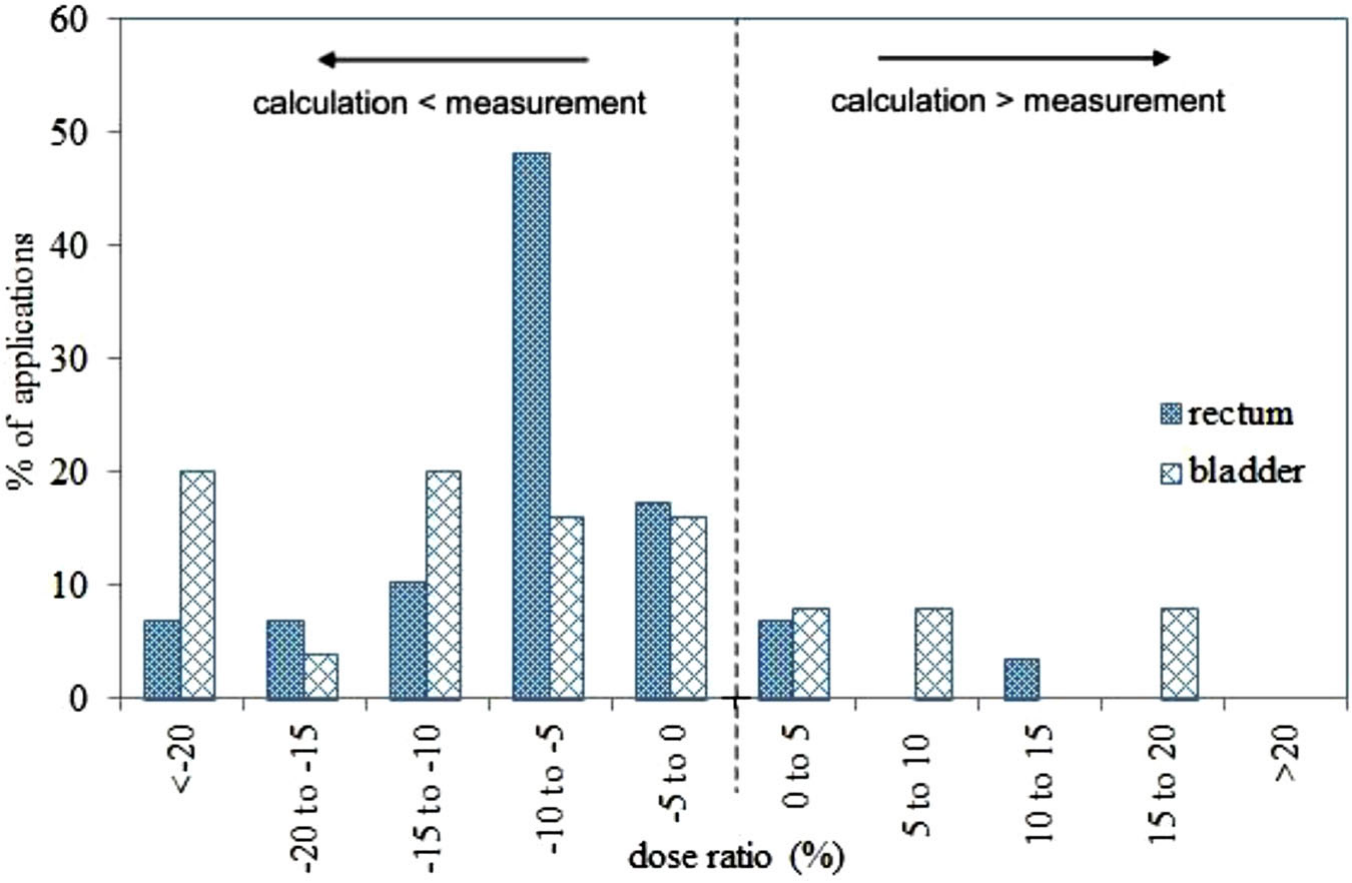
Histogram of the deviations between calculated absorbed doses by the treatment planning system (TPS) (=100%) and measured absorbed doses with the bead thermoluminescent dosimeters (TLDs) for 29 cases for the rectal absorbed dose and 25 cases for the bladder absorbed dose

The mean rectal and bladder measured absorbed doses were 3.0 ± 1.3 and 3.5 ± 1.2 Gy, respectively. The mean of relative absorbed dose difference (i.e., (calculated dose − measured dose)/calculated dose×100) for the rectum was −8.3% ± 19.5% (1SD) ranged from −86.7% to 50.9%. For 90% of cases, measured absorbed dose was higher than the calculated absorbed dose (Figure 2).

In addition, the relative difference for bladder calculated and measured absorbed dose ranged from −67.9% to 24.1%, with the mean of −7.2% ± 14.6% (1SD). In 76% of cases, higher measured absorbed doses were obtained than the calculated absorbed dose (Figure 2).

Data from two approaches for control point assignment (i.e., one per TLD or one per two adjacent TLDs) are presented in Table 3.

**TABLE 3:** Comparing the results of in vivo silica bead dosimetry in patients’ rectum and bladder with two methods of measurement point selection

Organ	Number of measured points	Measured absorbed dose (mean ± SD (Gy))	Calculated absorbed dose (mean ± SD (Gy))	*p*‐Value^a^	% Of absorbed dose difference (mean ± SD)
Rectum	40	3.0 ± 1.3	2.8 ±1.2	<0.001	−8.3 ± 19.5
	80	3.0 ± 1.3	2.8 ± 1.3		−7.7 ± 19.5
Bladder	5	3.5 ± 1.2	3.3 ± 1.1	<0.001	−7.2 ± 14.6
	10	3.5 ± 1.1	3.3 ± 1.1		−6.8 ± 14.1

^a^ Results of paired sample *t*‐test between the two methods of defining two adjacent TLDs.

**FIGURE 3:**
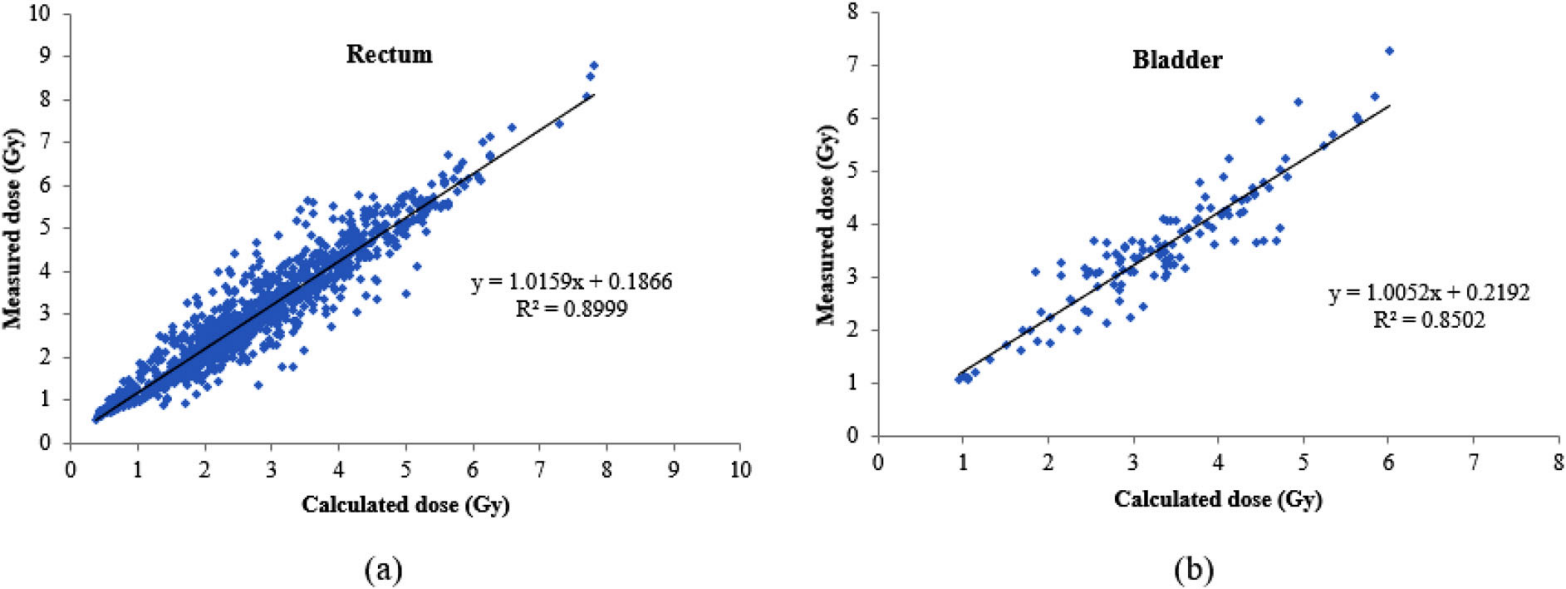
Scatter diagram presentation of the correlation between the measured and calculated point absorbed dose in (a) rectum and (b) bladder. The parameters for the linear regression, the slope, the intercept, and the correlation coefficient are specified

A scatter diagram of the measured and calculated absorbed doses for each organ is provided in Figure 3a,b. The linear regression curves and the slope, intercept, and the correlation coefficient was specified based on the data. The linear trend line equates to the ideal scenario where the measured and calculated absorbed dose points are equal. According to this figure, the correlation between calculated and measured absorbed doses for the rectum and bladder was 0.90 and 0.85, respectively.

**FIGURE 4:**
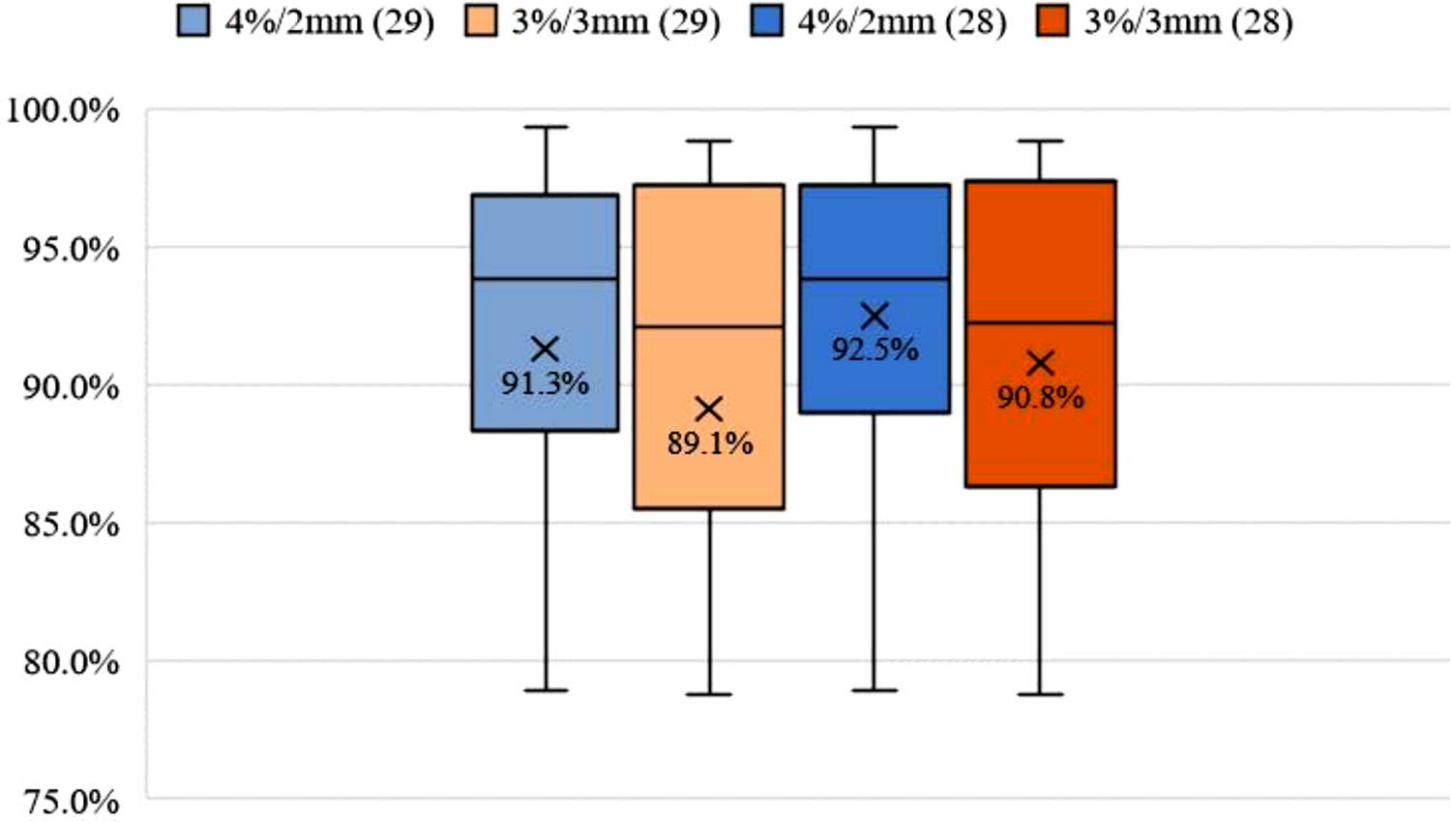
Box plots of gamma analysis were performed between measured and calculated rectal absorbed doses for 29 and 28 cases. Mean values have been indicated by cross at the box and whisker plots

Figure 4 provides box plots of gamma analysis performed between in vivo measured and calculated rectal absorbed doses for 29 and 28 cases. Data of the 3%/2‐mm criteria do not present in this figure to prevent the shape from getting crowded. Using gamma analysis to compare the calculated and measured absorbed dose matrix of considered cases, a mean pass rate of 82.3%, 89.1%, and 91.3% by 3%/2 mm, 3%/3 mm, and 4%/2‐mm criteria, respectively, were achieved. There was an outlier for which the rectal tubes had moved around 5 mm out of her body. This setup error resulted in a 20% relative difference in her mean rectal absorbed dose estimation. Therefore, this case was left out. By excluding this patient, the mean pass rate for the mentioned criteria was enhanced to 84.1%, 90.8%, and 92.5%.

## 4 DISCUSSION

The current research suggested a clinically practical patient‐specific protocol for IVD of OARs during the GYN BT by bead TLDs. The feasibility of the proposed protocol was tested and approved through a pilot study. It should be emphasized that the measured point absorbed doses can approximate the given absorbed doses to the internal walls of the rectum and bladder, but not an exact representation of their maximum received absorbed doses.

Higher measured absorbed doses than the calculated ones from the TPS (Figure 2) in most of the TLDs positions could be due to the TPS's dose calculation algorithm, which is based on the TG‐43 algorithm and water consideration of all surrounding media, including the beads. As was calculated before by Jafari et al., the CT Number of the glass beads was determined to be within the range of 800–1300 using a computerized tomography scanner, overlapping with the CT number of the cortical bone. Therefore, the glass beads were found to have a mass density of 2.09 ± 0.01 g/cm^3^, which is comparable to the 1.92‐g/cm^3^ mass density of cortical bone.

The results of Table 3 approved that there was no statistically significant difference (*p*‐value <0.001 using paired sample *t*‐test) between the two methods of defining two adjacent TLDs as one control point or selecting each bead as a separate control point on the TPS. Therefore, allocating a control point for two consecutive beads would not impact the in vivo organs absorbed dose measurements accuracy but keep the localization process easier and faster.

The measured and predicted absorbed dose to the OARs had excellent linear correlations, with *R* ^2^ = 0.90 and 0.85 for the rectum and bladder, respectively (Figure 3a,b). Waldhäusl et al. used diodes for IVD during cervical cancer BT. They obtained a correlation coefficient (*R* ^2^) of 0.66 for the rectum and 0.82 for the bladder, which has a good agreement with our results.^11^ They also found −31% to +90% (mean = 11%) and −27% to 26% (mean = 4%) differences between measured and calculated absorbed doses for rectum and bladder. In the current study, the mean percentage differences between calculated and measured rectal and bladder absorbed doses were −8.3% (ranging from −86.7% to 50.9%) and −7.2% (ranging from −67.9% to 24.1%), respectively, which are consistent with the other authors’ findings (Table 3). The high standard deviation of both of these studies could be justified by considering the high dose gradient of BT and high sensitivity to any intra‐fractional variations in detector location. Waldhäusl et al. concluded that a 2.5‐mm shift in the rectal probe and a 3.5‐mm shift in the bladder probe position would cause dose differences of more than 10%.^11^ Allahverdi et al. also compared TPS‐reported dose and the delivered dose to the rectum measured by diodes in GYN patients during HDR‐BT, which leads to a mean difference of 6.5% (ranging from −22% to +39%) in the rectum calculated and measured absorbed dose.^12^ Between the calculated absorbed doses to the rectum of 11 cervical cancer patients receiving HDR ^60^Co BT and their IVD results by diodes, Zaman et al. found a mean relative difference of 2.6% (ranging from −8.5% to 41.2%) and a correlation factor of *R* ^2^ = 0.88.^27^ Another study by Nose et al. investigated IVD of patients during HDR‐BT of their pelvic cancer utilizing radiophotoluminescence glass dosimeters. All of these studies determined that the significant difference between OARs’ calculated and experimentally measured doses was most likely due to the organs and applicators’ independent movements throughout the BT session.^28^

One of the essential factors in radiation dosimetry is the energy response of the dosimeter, mainly when its effective atomic number differs from the soft tissue. The beads show greater energy dependency in the lower energy range in which the photoelectric process is the dominant interaction. Despite their large effective atomic number and the change of the ^60^Co photon spectra to lower energies in these ranges, variation in beads’ TLD response is negligible (1%) at distances less than 12 cm from the ^60^Co source, according to Monte Carlo simulations.^16^ During the current study, no response correction was required because none of the bead TLDs was more than 12 cm away from the BT source.

Furthermore, the CTDIvol of the selected patients was 5.3–6.5 mGy, according to the CT scanner report. As a result of these CTDIvol and TLDs’ energy response curves at kV photon beams, a correction factor for TLDs with absorbed doses less than 1 Gy was applied to account for the absorbed dose from CT imaging. This correction factor was neglected for TLDs that were exposed to higher absorbed doses.^22^, ^29^

Eventually, the high standard deviation for the absorbed dose differences from IVD during the current study can be due to several factors, such as the BT steep dose gradient, inaccurate localization of the control points for TLDs in TPS, and uncertainties related to the intra‐fractional variations in the relative position of applicators to the OARs. Other potential factors contributing to the absorbed dose differences during GYN BT are variation in size, shape, and position of the OARs (e.g., the rectal peristaltic motion) and applicator geometrical shift during the gap time between the CT scanning and their treatment delivery.^9^, ^11^, ^27^

There is a lack of online organ motions verification during the treatment delivery in most of the BT departments. Therefore, intra‐fractional variations of organs and dosimeters’ position to the applicators are essential factors of BT uncertainties. New online MRI‐guided BT can be a good solution.^30^, ^31^

Some current integrated system for in vivo measurement has been launched to enable reporting online point absorbed dose during the BT procedure.^22^ However, there is still limited point absorbed dose measurements routinely.^32^ Array detectors (e.g., multiple diodes, MOSFET, and scintillation detector array) do not help overcome this limitation and are not applicable for use in BT IVD because of their large size.^33^, ^34^ Accordingly, the current project tried to use a large number of small bead TLDs for IVD of the rectum and passing from point to 3D dosimetry by performing gamma analysis between planned and measured absorbed dose maps. Therefore, the measurement methodology is more similar to external beam patient‐specific quality control for IMRT/VMAT dose delivery verification.

Determining a clinically acceptable passing rate for using gamma index in HDR‐BT is challenging. Pilot audits have indicated that evaluated gamma index of passing rates exceeding 95% is agreed, 90% is desirable, and 80% should be investigated, at 3%/3‐mm criteria.^26^ We evaluated the rectum's measured and calculated absorbed dose matrix using gamma analysis with different criteria and acquired different pass rates. By tabulating the results, we eventually found that 2%/2 mm is so strict for the proposed IVD, and thus it was omitted for reporting. Finally, considering Figure 4 results, choosing the pass rate of more than 85% is achievable in the clinical situation with 4%/2‐mm criteria.

The small size of silica bead TLDs and their dosimetric properties (e.g., linear dose–response, high sensitivity, absorbed dose rate and angular independence, extensive dynamic range, reusability, chemically inert nature, and low cost) have shown their potential for in vivo absorbed dose measurements and independent treatment delivery verification in GYN HDR‐BT.

The required time to string the annealed beads on a piece of plastic yarn and preparation of the rectal tube and bladder Foley was about 2 h. The time needed to allocate the TLDs on TPS depends on their number. In this study, it took about 1 h. Moreover, the required time to read the beads manually with the current commercial TLD readers was about 2 h/patient. The significant labor‐intensive and time‐consuming step of manually preparing and reading out the bead TLDs was the limitation of this study, which can be resolved with an automatic TLD reader.

## 5 CONCLUSION

A 3D IVD protocol employing bead TLDs was presented to measure absorbed doses delivered to the rectum and bladder during GYN HDR‐BT as a reliable PSQA method. 3D rectal in vivo absorbed dose measurements were performed. Differences between experimentally measured and planned absorbed dose maps in the form of gamma index were introduced, which can be considered an indicator of the need for corrective action or at least as a “considering level.”

## CONFLICT OF INTEREST

The authors declare that they have no known competing financial interests or personal relationships that could have appeared to influence the work reported in this paper.

## AUTHOR CONTRIBUTION

Ramin Jaberi, Maryam Moshtaghi, and Somayyeh Babaloui carried out the experiment. Somayyeh Babaloui and Zahra Siavashpour wrote the manuscript with support from Ramin Jaberi, Musa Joya, and Alireza Shirazi. Shakardokht Jafari helped supervise the project. Mohammad Hadi Gholami supports the data analysis.

## ETHICS STATEMENT

The Tehran University of Medical Sciences (TUMS) review board approved the study protocols (code of ethics: IR.TUMS.MEDICINE.REC.1396.4851). All patients were informed of the study's purposes, and written informed consent was obtained from all subjects before participation.
